# Investigation of DNA Damage and Apoptosis in Bovine Ocular Squamous Cell Carcinoma

**DOI:** 10.1002/vms3.70448

**Published:** 2025-06-26

**Authors:** Muhammet Bahaeddin Dörtbudak, Kerem Yener

**Affiliations:** ^1^ Faculty of Veterinary Medicine, Department of Pathology Harran University Şanlıurfa Turkey; ^2^ Faculty of Veterinary Medicine, Department of Surgery Harran University Şanlıurfa Turkey

**Keywords:** 8‐OHdG, bovine, carcinopathogenesis, CASP‐3, γ‐H2AX

## Abstract

Ocular squamous cell carcinoma represents the most prevalent form of cancer in cattle and exerts a deleterious effect on animal welfare and breeders. This study sought to elucidate the relationship between DNA damage and apoptosis with the aim of advancing our understanding of the pathogenesis of the disease. Nine bovine tissue samples with suspected ocular squamous cell carcinoma (OSCC) were examined histopathologically and immunohistochemically. The taken tissue samples, in histopathological examinations by haematoxylin‐eosin (HE), staining revealed the presence of neoplastic cells exhibiting various grades of differentiation. It observed the presence of polygonal or ovoid‐shaped cells with hyperchromatic nuclei and vesicular cytoplasm, as well as mitotic figures and bizarre giant cells. Islets and caudal extensions of the tumour cells were observed to contain keratin deposits. Immunohistochemical staining of OSCC tissues was performed using γ‐H2AX for DNA double‐strand breaks, 8‐OHdG for DNA oxidation and CASP‐3 biomarkers for nuclear apoptosis. The immunohistochemical examination demonstrated that the expression of 𝛾‐H2AX and 8‐OHdG was markedly elevated, while CASP‐3 expression was relatively diminished in aggressive tumours. In contrast, the opposite was observed in low‐grade tumours. This study is the first to report DNA double‐stranded breaks in eye cancers, and it has also shown that DNA helix breaks and guanine oxidation are effective in cancer pathogenesis. It has also been determined that apoptotic defence reactions due to DNA damage are reduced. In light of these findings, we made a significant contribution to the development of BOSCC carcinogenesis.

## Graphical abstract

This is the first study to report an increase in double‐stranded DNA breaks in BOSCC, an important tumoural disease of cattle.

In this study, where an increase in DNA oxidation was also shown, we determined that apoptotic defence reactions were reduced, contributing to the pathogenesis.

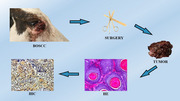

## 1 Introduction

Ocular squamous cell carcinoma (OSCC), also referred to as ocular carcinoma, has historically been the most prevalent neoplasm observed in cattle. The presence of ocular carcinoma has a detrimental impact on animal welfare and results in significant economic losses owing to decreased productivity. In addition to the hereditary factors of age, sex, race and hypopigmentation, various environmental factors, including diet, altitude, latitude and duration of exposure to sunlight, have been identified as effective in the aetiology of the disease. Furthermore, the disease has been linked to certain viral agents, including papillomaviruses and herpesviruses (Tsujita and Plummer 2010; Amritha et al. 2018; Choi et al. 2013; Karakurt et al. 2023). The tumour, which is formed by the transformation of plaque‐like proliferations originating from the stratum spinosum layer of the epithelium into papillomas, undergoes a series of changes over time, eventually developing into an in situ and invasive carcinoma (Song et al. 2012; Kapoor et al. 2020). These masses, which are typically cauliflower‐like in appearance and variable in size, demonstrate ocular and periocular localisation, including the cornea, eyelids, sclera and conjunctiva. Islets or cords of anaplastic cells with various grades of differentiation and keratin deposits are observed at the microscopic level. Histopathological examination is employed for the diagnosis of the disease. Surgical interventions, including expiration and exenteration, are typically used to treat this condition (Jhirwal et al. 2022; R. K. Sharma et al. 2024; Lakshmi et al. 2021).

H2AX is a variant of the H2A protein family, which forms a histone complex in nucleosomes. Following breaks in the DNA double‐strand, they are released via histone phosphorylation. This histone protein, which facilitates the repair of DNA breaks, is a crucial biomarker of DNA damage in contemporary studies (Scully and Xie 2013; Dörtbudak and Öztütk 2024). Guanine, which exhibits the lowest ionising property among the DNA components, is highly susceptible to oxidation. 8‐hydroxy‐2'‐deoxyguanosine (8‐OHdG), a byproduct of hydroxyl radical‐mediated oxidation at the eighth carbon atom of guanine, serves as a crucial biomarker for detecting oxidation‐induced DNA damage (Turan et al. 2023; Korkmaz et al. 2018). CASP‐3, a member of the cysteine–aspartic acid family, plays a pivotal role in apoptosis through the sequential activation of caspases. CASP‐3, which induces nuclear apoptosis in the absence of DNA damage, is also an important biomarker of apoptosis (Hussain et al. 2021; Cullen and Martin 2009).

The objective of this study was to investigate the relationship between DNA damage and apoptosis in OSCC tissues, with a particular focus on the correlation with tumour grade, as determined by histopathological examination. To this end, the expression intensities of current DNA damage and apoptosis biomarkers in cancerous tissues according to tumour grade were examined by immunohistochemistry.

## 2 Materials and Methods

### 2.1 Surgical Procedure

This study was completed in accordance with the procedures and principles of the Harran University Animal Experiments Local Ethics Committee (Session Number: 2024/004/09). A systematic clinical examination was conducted in patients presenting with lacrimal discharge and swelling in the eye and surrounding tissues at Harran University's Faculty of Veterinary Medicine's Department of Surgery. During the course of anamnesis, it was established that the lesions in the eyes of the animals initially manifested as lacrimal discharge, followed by the development of a lentil‐sized swelling in and around the eyelid. Subsequently, the swelling grew and became infected. Local tumour excision was performed in five cases, enucleation in two cases and exenteration in two cases. In the postoperative period, an eye pomade containing Thiocilline (2500 IU bacitracin and 25 mg neomycin sulphate; Ibrahim Hayri, Turkey) was applied for 5 days in cases where the local excision technique was employed. mg clavulanic acid, Zoetis, Turkey) was used, Synulox (7 mg amoxicillin and 1.75 mg clavulanic acid, B. Ingelheim, Turkey) was administered intramuscularly for 5 days, and Metacam (5 mg meloxicam, B. Ingelheim, Turkey) was administered as a single intravenous injection. Tissue samples obtained by surgical intervention were submitted to the Department of Pathology for histopathological examination.

### 2.2 Histopathological Examination

Following macroscopic examination, surgically removed masses were placed in 10% buffered formaldehyde. The tissues were subsequently washed and incorporated into a standard tissue follow‐up protocol. Subsequently, the tissues were embedded in a paraffin dispenser. Tissue sections of 5 µm thickness were obtained from each paraffin block using a rotary microtome (Leica RM2125, Germany). Following deparaffinisation and rehydration, tissue sections were stained with haematoxylin and eosin. The stained sections were dehydrated in alcohol, cleared in xylol and dripped with Entellan. Finally, a coverslip was placed on each slide. The slides were examined under a light microscope (Olympus BX53, Japan) and scored according to the severity of the histopathological findings as follows: absent (−), mild (+), moderate (++) and severe (+++) (Al‐Jameel et al. 2022). Furthermore, the tumours were graded based on the results of the histopathological examination in accordance with the criteria outlined in Table 1 (Carvalho et al. 2005; Sözmen et al. 2019).

**TABLE 1:** The histopathological grading of bovine ocular squamous cell carcinoma is discussed in two recent studies.

Tumour grade	Histopathological findings
G1	Large keratin pearls, enlarged tumour islands, substantial intercellular bridges, good squamous differentiation
G2	Small to medium‐sized keratin pearls, smaller islands, moderate keratinisation and differentiation, squamous differentiation, increased number of poorly differentiated cells
G3	Very little sign of keratinisation, individual cell keratinisation, few small tumour islands, poor cellular differentiation

### 2.3 Immunohistochemical Staining

Tissue sections mounted on adhesive slides were incubated in an oven for a designated period, after which they were deparaffinised and rehydrated. To inactivate endogenous peroxidase, the tissues were incubated in 3% hydrogen peroxide for 30 min, washed with phosphate‐buffered saline (PBS), boiled, and cooled three times in citrate buffer to release antigens. The specimens were subjected to a washing procedure utilising PBS. A protein block was added to the tissues to prevent non‐specific antigen binding and incubated for 20 min. Subsequently, primary antibodies targeting 𝛾‐H2AX, 8‐OHdG and CASP‐3 were introduced to the tissues and incubated at +4°C for 16 h. The secondary antibody was subsequently added to the tissues, which had been washed with PBS, and incubated for a period of 20 min. Subsequently, the tissues were washed with PBS and incubated for 20 min with a streptavidin peroxidase conjugate. Once the optimal colour gradient had been achieved by introducing 3‐3' diamino benzidine (DAB) chromagen into the tissues that had been washed with PBS for the final time, a further washing step was performed with distilled water. Subsequently, background staining with Mayer's haematoxylin was conducted. The tissues were subsequently washed with distilled water, dehydrated in alcohol, cleaned in xylol and coverslipped. The tissues were examined under a light microscope (Olympus BX53, Japan), and according to the severity of immune reactions as absent (−), mild (+), moderate (++) and severe (+++). (Ciaputa et al. 2019; Carvalho et al. 2005).

### 2.4 Statistical Analysis

The data were analysed using the statistical software package SPSS (22.1). The Kruskal–Wallis test was employed to assess the impact of tumour grades on both histopathological and immunohistochemical findings, while the Mann–Whitney *U* test was used for comparisons between the two groups. The Friedman test was employed to compare ordinal data across all cases, with the aim of evaluating the differences between histopathological findings. The relationships between the biomarkers in the immunohistochemical examinations were evaluated using Spearman's correlation analysis. The results of the statistical analyses were deemed statistically significant at *p* < 0.05.

## 3 Findings

### 3.1 Clinical Findings

In this study, nine cattle diagnosed with BOSCC based on the results of histopathological examination were used. Each animal exhibited lesions in one eye. The cases were of the Simmental breed (three females and two males) and the Holstein breed (three males and one female), with an age range of 3–7 years. Lesions were observed in both eyes, five of them localised in the left eye and four in the right eye. Only one was observed in the lateral canthus, whereas the others were equally distributed between the corneal conjunctiva and eyelid regions. The degree of lacrimation observed in the affected eyes was contingent on the macroscopic characteristics and infection status of the masses present. The lesions exhibited a range of sizes; it has complete vision loss in three animals and visual impairment in the remainder. The study revealed that none of the nine cattle evaluated exhibited any signs of metastasis, recurrence or other complications within the 6‐month observation period.

### 3.2 Macroscopic Finding

**FIGURE 1:**
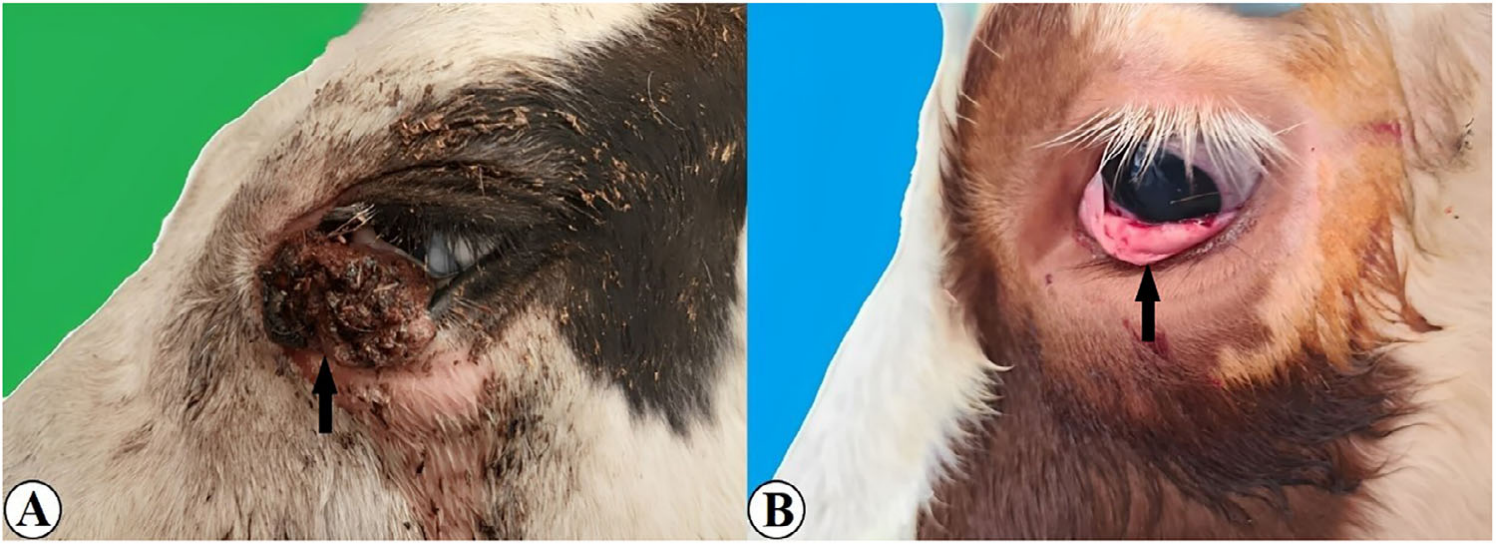
Macroscopic view of BOSCC tumour tissue (A) Ulcerative tumour mass localised in the inferior conjunctiva and third eyelid in Holstein cattle (B) Tumour mass localised in the inferior conjunctiva in Simmental cattle.

Macroscopic examination revealed that the tumours were typically cauliflower‐like in appearance, irregular contours, a stalked or sessile growth pattern, and hard, fragile consistency. They were observed to have a grey–pinkish or brown–grey colouration. On the cross‐sectional surfaces of these pathological formations, which exhibited a mean diameter of 2–10 cm, the presence of epithelial growths with a grey–white colour extending towards the depths was discerned. In some cases, the tumour tissues were infected, and purulent, haemorrhagic, necrotic or ulcerative foci were observed on them or surfaces (Figure 1A,B). Data regarding the breed, age and sex of the animals in which the tumours were observed, along with the localisation and macroscopic characteristics of the tumours, are presented in Table 2.

**TABLE 2:** Clinical and macroscopic findings in tumour cases.

Case no.	Folk	Age	Gender	Localisation	Size	Macroscopic character
1	Simmental	5	F	Right corneal conjunctiva	5.6.5	Verrucosis
2	Holstein	4	M	Left 3rd eyelid	3.5.4	Multi‐plate
3	Simmental	7	M	Left corneal conjunctiva	9.7.10	Verrucous, haemorrhagic–ulcerative
4	Simmental	3	F	Right corneal conjunctiva	2.3.2	Nodular, necrotic
5	Holstein	5	M	Right superior eyelid	8.5.7	Compacted plaque, lobular purulent
6	Holstein	5	M	Left corneal conjunctiva	7.6.4	Multi‐plate, ulcerative
7	Simmental	6	M	Left 3rd eyelid	9.10.7	Verrucous, necrotic–ulcerative
8	Holstein	4	F	Left lateral conthus	2.5.3	Nodular, ulcerative
9	Simmental	6	F	Right 3rd eyelid	6.5.7	Nodular

### 3.3 Histopathological Findings

**FIGURE 2:**
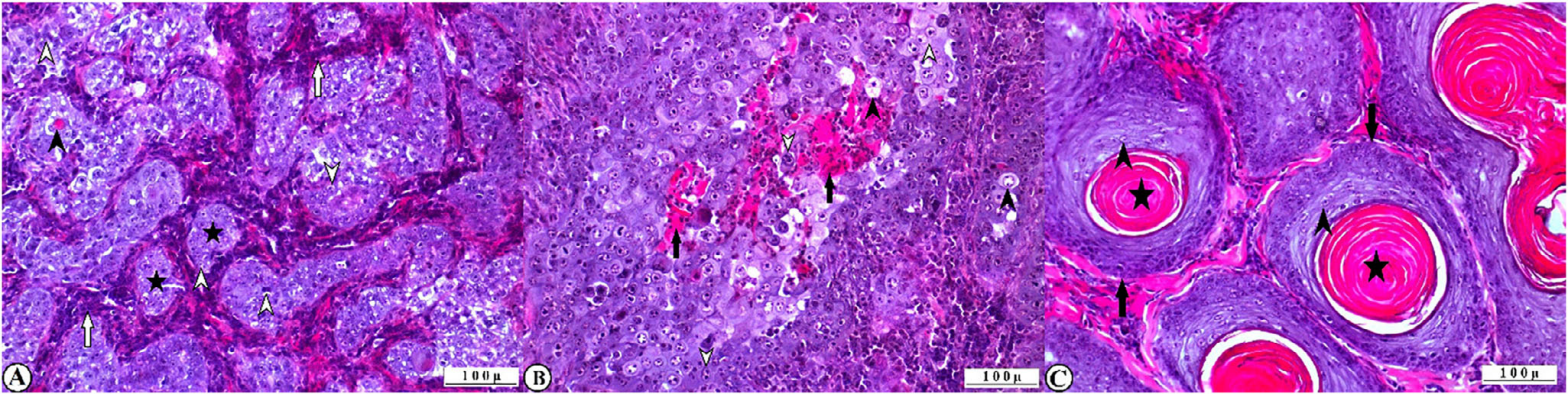
Histopathological appearance of BOSCC tissue, HE, X200 (A) G3 grade tumour tissue specimen showing poorly differentiated neoplastic cells with dyskeratotic (arrowhead) and mitotic figures (hollow arrowhead), small tumour islands (stars), inflammatory cell infiltration in fibrovascular stroma (hollow arrows). (B) Differentiated cells with mitotic figures (hollow arrowhead) and vesicular nucleus (arrowhead), intercellular keratin deposits (arrows) in G2 grade tumour tissue sample. (C) Large lamellar keratin pearl (stars), large tumour islands (arrows) and well‐differentiated neoplastic cells (arrowheads) in a G1 grade tumour specimen.

Microscopic examination of the tumour tissue revealed many anaplastic cells with various grades of differentiation. Depending on the degree of aggression, these cells showed polygonal or ovoid pleomorphism with hyperchromatic or pyknotic vesicular nuclei and pale eosinophilic cytoplasm. These cells showed dyskeratosis, anisocytosis, anisonucleosis and an increase in the nuclear/cytoplasmic ratio in favour of the nucleus. Numerous mitotic figures and sometimes bizarre giant cells were observed. These neoplastic cells were localised as islets or cords, surrounded by the stroma. In the centre of these cell islets, keratin deposits known as keratin or keratin pearls were observed, sometimes in large lamellae, rarely containing chromatin. In poorly differentiated anaplastic epithelial cells with poor squamous differentiation, keratinisation was restricted to cell clusters, whereas well‐differentiated atypical squamous cells showed large keratin globes and cords extending between these structures. Poorly differentiated cell masses invaded the stroma as pseudocordons and fibrous connective tissue proliferated between these cells. Severe necrosis, inflammatory cell infiltration and vascular changes were observed in the epidermal layer of cases that developed ulcerations with secondary infection (Figure 2A–C).

The tumour samples were graded as in previous studies, considering the severity of differentiation in tumour cells, keratin production and size of the tumour islands, which indicate the aggressiveness of the tumour. Aggressive G3‐grade tumours were observed in five samples. These tumours showed poor keratinisation, poor differentiation and small tumour islands. Two specimens showed G2 grade tumours with better differentiation than G3, more prominent tumour islands, and keratin production. The other two specimens showed G1 grade tumours with better differentiation close to the cell of origin, large keratin deposits and large tumour islands surrounded by stroma. The histopathological findings of the tumours are shown in Table 3.

**TABLE 3:** Evaluation of histopathological findings in tumour cases.

Case no.	Differentiation	Mitotic figure	Keratinisation	Invasion	Inflammation
1	++	+	++	+	−
2	+++	+	++	−	+
3	+	++	+	++	+
4	+++	++	+++	+	−
5	+	++	+	+++	++
6	+	+++	+	++	+++
7	+	+++	++	++	++
8	++	++	+	−	+
9	+	+++	+	+++	+++

Statistical analysis revealed a significant difference among the histopathological findings (*p* = 0.008). Specifically, mitotic activation emerged as a key parameter in tumour characterisation, suggesting its pivotal role in distinguishing between different tumour grades or types (*p* = 0.033).

### 3.4 Immunohistochemical Findings

**FIGURE 3:**
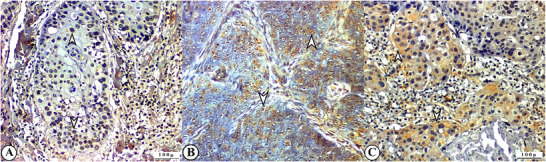
Immunohistochemical findings of OSCC tissue, IHC, X200 (A) 𝛾‐H2AX expression in tumour cells (arrowheads) (B) 8‐OHdG expression in tumour cells (arrowheads) (C) CASP‐3 expression in tumour cells (arrowheads).

Immunohistochemical examination revealed 𝛾‐H2AX, 8‐OHdG and CASP‐3 immunopositivity in all cases (Figure 3A–C). Intense 𝛾‐H2AX and 8‐OHdG expression was observed in the cytoplasm and nuclei of poorly differentiated neoplastic cells, especially in small tumour islands in tumour aggressive samples. CASP‐3 expression was low in these samples. Immunopositivity was seen in the cytoplasm of bizarre giant cells, mitotic figures and hyperchromatic neoplastic cells in tumour islands and cords, sometimes in granular form. In low‐grade tumours, the immunoreactivity for H2AX and 8‐OHdG was weak, and the expression of CASP‐3 was strong. Immunohistochemical findings related to tumour grade and severity of H2AX, 8‐OHdG and CASP‐3 expression are shown in Table 4.

**TABLE 4:** Tumour grade and immunohistochemical findings in tumour cases.

Case no.	Tumour grade	𝛾‐H2AX expression	8‐OHdG expression	CASP‐3 expression
1	G2	**++**	**+++**	**+**
2	G1	**+**	**++**	**+++**
3	G3	**+++**	**+++**	**+**
4	G1	**+**	**+**	**++**
5	G3	**+++**	**++**	**+**
6	G3	**++**	**+++**	**+**
7	G3	**+++**	**+++**	**+**
8	G2	**++**	**+**	**+++**
9	G3	**++**	**++**	**+**

Statistical analysis of the immunohistochemical results showed no significant differences in H2AX and 8‐OHdG expression across groups (*p* = 0.36), whereas a significant difference was observed in CASP‐3 expression (*p* = 0.034). In addition, a statistically significant negative correlation was observed between markers of DNA damage (8‐OHdG) and apoptosis (CASP‐3), indicating that increased DNA damage was associated with decreased apoptosis.

As a result, no statistically significant difference was observed between tumour grades in terms of γ‐H2AX and 8‐OHdG expression levels (*p* = 0.36 for both). However, a statistically significant difference was found in CASP‐3 expression (*p* = 0.034), especially between G1 and G2 and G1 and G3 (*p* < 0.05). The statistical results are presented in Table 5.

**TABLE 5:** Summary of statistical comparison of biomarker expressions between tumour grades.

Biomarker	Tumour grade trend (median, IQR)	Overall *p* value (Kruskal–Wallis)	Pairwise significant differences (Mann–Whitney *U*)
γ‐H2AX	G1: + (1–2) G2: ++ (2–3) G3: +++ (3–4)	0.36	No statistically significant pairwise differences
8‐OHdG	G1: + (1–2) G2: ++ (2–3) G3: +++ (3–4)	0.36	Not significant in pairwise comparisons
CASP‐3	G1: +++ (2–3) G2: ++ (2–3) G3: + (1–2)	0.034	G1 vs. G2 (*p* = 0.03) G1 vs. G3 (*p* = 0.01)

## 4 Discussion

BOSCC is a malignant ocular tumour arising from squamous epithelial cells and is common in cattle. BOSCC causes loss of productivity in animals and is therefore an economic loss. Genotypic characteristics and environmental factors have been reported to play a role in disease aetiology. It has been reported that Simmental and Holstein cattle are more susceptible to this type of cancer (Tsujita and Plummer 2010; Ceylan et al. 2012). In this study, this tumour was found in Simmental and Holstein cattle. In addition, the duration of exposure to sunlight is an important environmental factor, and the animals from which the study materials were obtained were frequently exposed to sunlight in the region.

Although macroscopic examination provides a clue for the diagnosis of the disease, it is not the only way to make a definitive diagnosis. Macroscopic examination revealed tumour masses of varying sizes and macroscopic appearances in the eye and surrounding tissues, depending on the aggressiveness of the cancer. These tumours, sometimes characterised by simple plaque‐like tissue growth, may be verrucous and nodular. Necrotic, ulcerative and purulent changes have also been reported to occur on the surfaces of infected tissues (V. Kumar et al. 2023; S. Sharma et al. 2020; Karakurt et al. 2021). Consistent with previous studies, the tumour masses in this study were generally cauliflower‐like, dark in colour, hard in consistency and of fragile structure.

Histopathological examination is useful in determining tumour prognosis and definitive diagnosis. Microscopic examination revealed anaplastic and mitotic figured cells with various grades of differentiation from the normal cell architecture, as in many cancers (Ahmed and Sözmen 2020; Kim et al. 2016). In the histopathological examination of BOSCC, it was found that the tumour cells showed hyperchromasia and ovoid or polygonal pleomorphism in the nuclei, and their cytoplasm was eosinophilic and contained large vesicles. These cells can be seen as mitotic figures or bizarre giant cells. One of the specific microscopic findings of BOSCC is keratin pearl, which may be intracellular and have been observed in a broad lamellar structure. In some BOSCC studies, the differentiation of neoplastic cells, keratinisation, and invasion of tumour clusters have been considered important criteria for grading cancer severity (Al‐Jameel et al. 2022; Carvalho et al. 2005; Ceylan et al. 2012; Kutlu et al. 2022). In this study, where microscopic findings were consistent with those of previous studies, the tumours were graded using similar criteria. Accordingly, G1, G2 and G3 grade tumours were found in five, two and two cases, respectively. In addition, mitotic figures were found to be a prominent parameter in histopathological evaluations.

As with many diseases, the fight against cancer can only be achieved by understanding its pathogenesis. Studies on the pathogenesis of BOSCC are limited. In this study, the expression of some biomarkers that indicate DNA damage was monitored to contribute to the pathogenesis of BOSCC. One such biomarker is γ‐H2AX; however, its use in animals is limited (Srivastava et al. 2009; Solier and Pommier 2014). Nakamura et al. (2017) used a γ‐H2AX biomarker to detect DNA breaks in bovine lymphocytes following the Fukushima disaster. Fradet‐Turcotte et al. (2011) showed that papillomaviruses cause DNA damage via γ‐H2AX expression. Toyoda et al. (2015) showed γ‐H2AX expression in bladder cancer, and Dörtbudak and Öztürk (2024) showed γ‐H2AX expression in goat intestinal tuberculosis. There have been no studies finding, in the literature review, γ‐H2AX expression, which indicates DNA breaks in BOSCC. In this study, γ‐H2AX expression was found to increase with cancer severity in BOSCC tissue.

Another biomarker investigated for DNA damage is 8‐OHdG, which is currently the preferred biomarker for detecting DNA oxidation. 8‐OHdG expression has been observed in many human cancers and experimental models (A. Kumar et al. 2012; Kubo et al. 2014). Karakurt et al. (2024) reported 8‐OHdG expression in their study on oxidative and nitrosative stress in bovine OSCC. This study found that 8‐OHdG expression was present in all tissues of BOSCC and that 8‐OHdG expression increased with tumour aggressiveness.

Apoptosis, the controlled death of cells, is affected by many factors, particularly by DNA damage. Several biomarkers have been used to determine apoptosis, which has been the subject of many cancer studies. In apoptosis, DNA fragmentation into nucleosomal subunits is caused by the inhibition of caspase‐activating DNase (ICAD) by caspase‐3 and the activation of caspase‐activating DNase (CAD). CASP‐3, which plays a key role in chromatin condensation, DNA fragmentation, and nuclear apoptosis, also contributes to the surveillance of pathological events (Majtnerová and Roušar 2018; Yap et al. 2020; Zhao et al. 2023). Kapoor et al. (2020) reported caspase‐3 expression in BOSCC. Yildız and Karakurt (2022) found that caspase‐3 expression decreased with tumour severity in BOSCC studies. It is predicted that this is due to the loss of balance between cell division and death, that is, the lack of signals required for controlled death. In this study, in line with previous studies, CASP‐3 expression decreased with increasing tumour severity. This suggests that apoptosis suppresses malignancy, whereas apoptosis is insufficient in aggressive tumours.

## 5 Conclusion

In this study, the relationship between tumour severity, DNA damage, and apoptosis was investigated to contribute to the pathogenesis of BOSCC, which is an important health problem in cattle production. DNA double‐strand breaks and nucleobase oxidation increased in aggressive cases with severe tumours, whereas apoptosis was suppressed. The opposite situation was observed for weak tumours. This study is the first to demonstrate double‐strand breaks with γ‐H2AX expression in BOSCC and to highlight the role of DNA damage and apoptosis in the pathogenesis of the disease and the studies that need to be undertaken to treat the disease.

## Author Contributions

**Muhammet Bahaeddin Dörtbudak**: conceptualisation, writing – original draft, methodology, visualisation, writing – review and editing, project administration, resources. **Kerem Yener**: investigation, validation, formal analysis, software, data curation.

## Conflicts of Interest

The authors declared no conflicts of interest.

## Ethics Statement

The study was approved by the Harran University Animal Experiments Local Ethics Committee (Protocol Number: 2024/004/09).

## Peer Review

The peer review history for this article is available at https://www.webofscience.com/api/gateway/wos/peer‐review/10.1002/vms3.70448.

## Data Availability

All data and materials of the study are available in contact with the corresponding author.
